# A Novel Computer-Vision Approach Assisted by 2D-Wavelet Transform and Locality Sensitive Discriminant Analysis for Concrete Crack Detection

**DOI:** 10.3390/s22228986

**Published:** 2022-11-20

**Authors:** Vahidreza Gharehbaghi, Ehsan Noroozinejad Farsangi, T. Y. Yang, Mohammad Noori, Denise-Penelope N. Kontoni

**Affiliations:** 1 School of Civil Engineering, University of Kansas, Lawrence, KS 66045, USA; 2 Faculty of Civil and Surveying Engineering, Graduate University of Advanced Technology, Kerman 7631818356, Iran; 3 Department of Civil Engineering, The University of British Columbia (UBC), Vancouver, BC V6T 1Z4, Canada; 4 Department of Mechanical Engineering, California Polytechnic State University, San Luis Obispo, CA 93407, USA; 5 School of Civil Engineering, University of Leeds, Leeds LS2 9JT, UK; 6 Department of Civil Engineering, School of Engineering, University of the Peloponnese, GR-26334 Patras, Greece; 7 School of Science and Technology, Hellenic Open University, GR-26335 Patras, Greece

**Keywords:** deep learning, FastCrackNet, crack classification, noisy data, wavelet, locality sensitive discriminant analysis

## Abstract

This study proposes FastCrackNet, a computationally efficient crack-detection approach. Instead of a computationally costly convolutional neural network (CNN), this technique uses an effective, fully connected network, which is coupled with a 2D-wavelet image transform for analyzing and a locality sensitive discriminant analysis (LSDA) for reducing the number of features. The algorithm described here is used to detect tiny concrete cracks in two noisy adverse conditions and image shadows. By combining wavelet-based feature extraction, feature reduction, and a rapid classifier based on deep learning, this technique surpasses other image classifiers in terms of speed, performance, and resilience. In order to evaluate the accuracy and speed of FastCrackNet, two prominent pre-trained CNN architectures, namely GoogleNet and Xception, are employed. Findings reveal that FastCrackNet has better speed and accuracy than the other models. This study establishes performance and computational thresholds for classifying photos in difficult conditions. In terms of classification efficiency, FastCrackNet outperformed GoogleNet and the Xception model by more than 60 and 80 times, respectively. Furthermore, FastCrackNet’s dependability was proved by its robustness and stability in the presence of uncertainties produced by network characteristics and input images, such as input image size, batch size, and input image dimensions.

## 1. Introduction

The majority of civil engineering facilities were built decades ago and have structural flaws owing to deteriorating materials, fire, chemical attacks, impacts, and seismic loads. According to the American Society of Civil Engineers (ASCE), 56,000 bridges are structurally defective and require 123 billion dollars for repair [1]. The collapse of the Minnesota I-35W bridge [2] and the 16-story Plasco building in Tehran are examples of disasters caused by ignoring structural conditions [3].

Structural health monitoring (SHM) provides innovative solutions for the monitoring and identification of various damages in engineering infrastructure, from large dams and long roads to small residential buildings. Here, damage is defined as any changes in the material and/or geometric properties, which can be reflected as concrete cracks, spalling of concrete cover, steel corrosion, fatigue cracks, or buckling of reinforced rods. Routine visual inspection by a trained specialist, along with conducting nondestructive tests (NDTs), such as penetrant testing, eddy-current, magnetic particle testing, ultrasonic and X-rays, and CT scans, are common and initial approaches for condition assessment [4]. However, SHM provides different contact or non-contact solutions that are automated, economical, and with higher accuracy and the least amount of human intervention.

For decades, a wide number of contact-based condition assessment approaches have been established by researchers. For instance, vibration-based damage detection (VBDD) methods use natural frequency, mode shapes, and frequency response functions. A large number of these methods require the attachment of various types of sensors for recording displacement, strain, or acceleration. While VBDD techniques can be useful to evaluate both the global behavior of structure and conditions of components locally, embedding numerous sensors and interrupting the servicing of structure puts a limit on them in practical engineering applications when time and money are critical manners.

Recent advancements in sensors, optics, computer capabilities, and memory capacity have made it easier to do a huge number of computations, which has led to a quick revolution in image processing algorithms. In both traditional vision-based systems for edge detection, which need manual feature extraction and human vision to evaluate the results, or in advanced computer vision techniques employing deep learning, these accomplishments are evident.

Civil engineers have performed extensive research to create and modify computer-vision methods for monitoring and investigative purposes. Additionally, vision-based techniques, when coupled with cameras and drones, provide the potential for quick and automated infrastructure inspection and condition monitoring. Deep learning has reinforced and enhanced traditional vision-based damage diagnosis for a wide scope of visual defects, including cracks, spalling, and corrosion [5]. 

Modern deep learning-based computer vision techniques are used for damage detection in three categories: object detection, image classification, and semantic segmentation. Deep learning models encompass long short-term memory (LSTM) [6], convolutional neural networks (CNNs) [7], deep belief networks (DBNs) [8], denoising auto-encoders (DAEs) [9] and recurrent neural networks (RNNs) [10]. Among the methods described, CNNs play an important role in all triple groups of computer vision.

Training a deep learning architecture from scratch demands a vast quantity of data and, as a result, increased processing resources. Transfer learning provides a solution for scientists. This means that the model may be used to solve a new problem by transferring existing knowledge, and the approach can be employed to tackle a new problem by utilizing a pre-trained model. Transfer learning is divided into two key stages: a pre-trained phase for gathering information from multiple activities and a fine-tuning step for applying the acquired knowledge to the target tasks. Transfer learning addresses the gap left by the requirement for large amounts of data, and as a result, it has been applied in a variety of computer vision application fields. Accordingly, a set of CNNs is trained using the ImageNet dataset [11]. As a result, the ImageNet dataset has been used to train a set of CNNs [12]. Several computer vision tasks, including image segmentation [13,14,15], object detection [16,17,18], image classification [11,19], and captioning [20,21], have been studied using pre-trained deep learning models.

Multiple pre-trained networks, including GoogleNet, AlexNet, VGG16, Xception, InceptionV3, and Resnet50, have reached state-of-the-art accuracy in various image identification problems. In this regard, CNN architectures have been extensively utilized for crack classification [22,23], pavement crack recognition [24,25], and crack segmentation [14,26].

In order to precisely evaluate surface imperfections on bridges made of cement concrete, Zhu and Song [19] upgraded VGG-16’s framework. Before being preprocessed with morphology-based weight adaptive denoising, the photos were segregated into a training and test set. To validate its performance, the enhanced VGG-16 was compared to conventional shallow neural networks (NNs) such as the backpropagation neural network (BPNN), support vector machine (SVM), and deep CNNs such as AlexNet, GoogleNet, and ResNet using the same dataset of surface defects on cement concrete bridges. The findings demonstrate that the suggested technique can extract multi-layer features from surface defect data while emphasizing edges and textures.

Wu et al. [27] presented GoogleNet and Inception V3 as a crack-detecting method based on a convolutional neural network. They demonstrated that the crack dataset retrained the pre-trained GoogleNet and Inception V3 model to better detect crack images. 

Islam et al. [23] developed a transfer learning approach based on CNNs to recognize concrete surface cracks from images. The transfer learning technique used in this study employs four existing deep learning models named ResNet18, DenseNet161, VGG16, and AlexNet, with pre-trained weights.

Deep learning has previously been used to classify images. Few researchers have focused on the negative effects of noise [28]. Numerous papers have created algorithms to improve image quality rather than classify images. Another disadvantage of utilizing pre-trained models is the time-consuming training process, which is especially difficult for larger architectures that require a considerable amount of memory. As a result, there is a research gap where approaches to correctly classify concrete cracking data with the presence of image noise in a quicker fashion are required.

To fill the previous gaps, the present authors propose a computationally efficient approach for classifying concrete cracks under unfavorable operational and environmental situations, such as noise and network uncertainties. To that end, this work investigates the potential of signal transformations and feature conditioning methods in order to design a novel technique capable of achieving the following objectives:

- Enhancing the performance of crack classification compared to pre-trained CNNs.
- Stable in terms of model parameter uncertainty.
- Keeping a high level of robustness in unfavorable imaging situations.
- Potential to adapt adjustments in the quantity of training images and image size.
- Benefit from high speed to lower the time of computation.

As a result, the standard classification strategy based on deep learning networks is established as the reference method, and the new approach outlined in this paper is compared to this reference.

Consequently, the remainder of the paper is structured as follows. First, the three major calculation steps of FastCrackNet are described along with an overview of the theories underpinning the proposed technique. Next is a description of the reference pre-trained CNN models. The image dataset used for comparing the efficiency of the methods is provided in the following section. The next part compares the classification results of cracks under various iterations to those obtained by typical CNNs. The subsequent sections evaluate the algorithm’s robustness and stability under various types of uncertainty. The conclusion section concludes with a discussion of the research findings.

## 2. Materials and Methods

Computer vision-based SHMs are becoming more prevalent as a result of their quick and easy installation in many engineering projects. However, in real-world applications, stability and robustness must be addressed. The other argument is about deep learning models, which are vital in computer vision. However, the processing capabilities and complexity of deep learning networks may impose constraints when using embedded single-board GPUs with inferior performance and power supplies. As a result, the emphasis in this novel methodology is on demonstrating an efficient computer-vision algorithm that can withstand adverse environmental conditions via a swift and non-complex network design.

**Figure 1:**
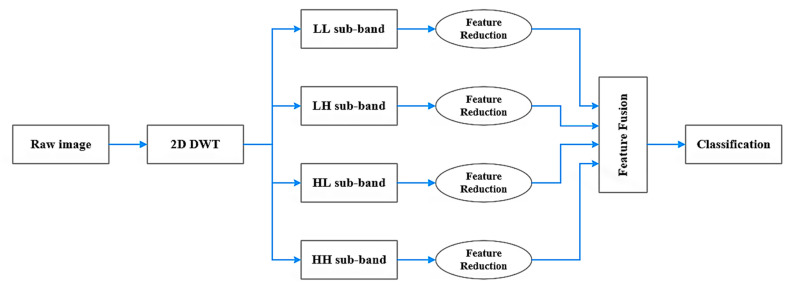
FastCrackNet Architecture.

To that purpose, three primary FastCrackNet components are given initially: image transformation, feature reduction, and classification network. The FastCrackNet architecture is depicted in the diagram of Figure 1. The suggested method is compared to common pre-trained CNN models in terms of performance and speed in the next sessions.

### 2.1. Step One: Image Transformation Using Wavelet

Herein, a new feature space is generated with the aid of time–frequency transformation. There are numbers of transformations in frequency and time–frequency domains, such as Fourier transformation and fast Fourier transformation [29], short-time Fourier transform [30], Hilbert transform [31], and Stockwell transform [32]. One of the most common approaches is wavelet transformation, used by several researchers in the realm of computer vision and image processing [5].

Wavelet methods are particularly well-suited to situations where scalability and tolerable deterioration are critical concerns. The wavelet transform breaks down a signal into a series of basic functions. Wavelets are created by scaling and translating a single prototype wavelet φ known as the mother wavelet [33].

(1) $$\varphi \left(s,\tau \right)=\frac{1}{\sqrt{s}}\varphi \left(\frac{t-\tau }{s}\right)dt\;$$

where s and τ are scale and transition factors, respectively, and $\sqrt{s}$ is for energy normalization at different scales. While continuous wavelet is highly redundant, discrete wavelets have solved this issue by scaling and transitioning in discrete intervals. The discrete wavelet for ’I’ level is presented in the equation below:

(2) $$\varphi \left(2^{j}t\right)=\underset{k}{\sum }\gamma_{I+1}\left(k\right)\theta \left(2^{I+1}t-k\right)t$$

where θ is the scaling function and γ shows the wavelet filter.

The wavelets are sampled at distinct intervals in the discrete wavelet transform. The normal image space, expressed as a pixel matrix, is referred to as the spatial domain. Transformation algorithms in this area operate directly on image pixel values. The frequency domain is concerned with the rate at which these spatial domain pixel values change. Frequency refers to the rate at which an image’s color components change. DWT provides simultaneous spatial and frequency resolutions. Combining an analytical filter bank with a decimation method, DWT is utilized to break down a picture into discrete sub-bands. Two major frequency components exist: high-frequency components that relate to image edges and low-frequency components that correspond to smooth areas [34]. Combining two separate 1D transformations yields the 2D transform.

**Figure 2:**
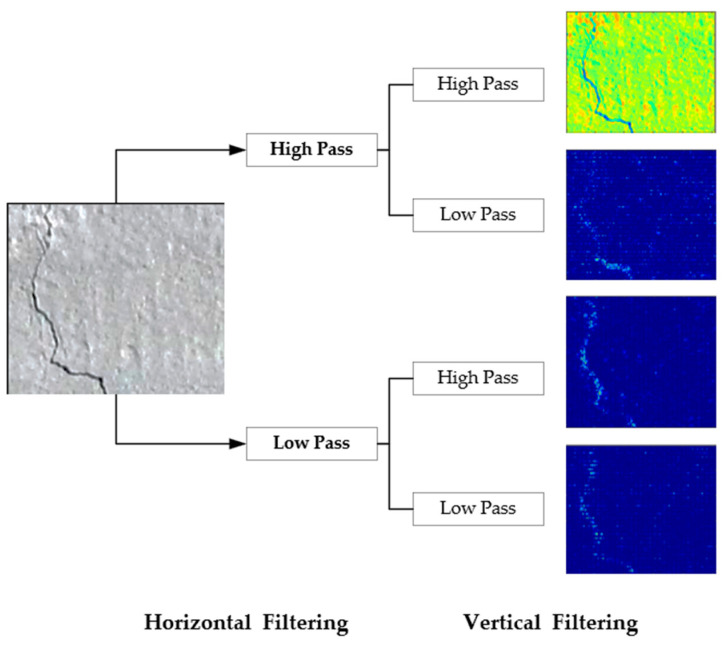
Image decomposition using 2D-DWT.

In 1D-DWT, approximation coefficients include low-frequency data, whereas detail coefficients include high-frequency information. The approximation illustrates the general trend of pixel values as well as the specifics, such as the horizontal, vertical, and diagonal components. Two-dimensional DWT divides the input picture into four distinct sub-bands, as seen below [35] (See Figure 2):

- LL: low-frequency components oriented horizontally and vertically.
- LH: low-frequency components oriented horizontally and vertically.
- HL: both the horizontal and vertical directions include components with a high frequency.
- HH: both the horizontal and vertical directions include components with a high frequency.

Daubechies [35] proposed a new mother wavelet (Daub). It is an orthogonal wavelet with scaling and wavelet function coefficients that show vanishing moment numbers. Depending on the sequence of the filter coefficients, other kinds of transformations using Daubechies families have been defined, such as Daub1 and Daub2. In this area, Daub4 consists of four scaling and wavelet coefficients. The Daub4 wavelet is popular due to its smoother behavior and the small number of coefficients [36]. Accordingly, Daub4 is used as the mother wavelet in the current method to extract features from concrete images.

### 2.2. Step Two: Feature Reduction

When presented with various characteristics that are not essential for predicting the intended output, supervised machine learning practical approaches lack efficacy (prediction accuracy). The extraction of a limited number of essential features is a high priority in computer vision, machine learning, knowledge identification, and pattern recognition. Using dimensionality reduction methods is a common strategy for overcoming this difficulty.

In this study, we employed a method of linear discriminant analysis termed Locality Sensitive Discriminant Analysis (LSDA). When there is a shortage of training data, the local structure is often more important than the global configuration for discriminant analysis. LSDA finds a projector that optimizes the gap between data points from different classes in each local region by identifying the local manifold structure. The data points are precisely mapped into a subspace, where neighbors with the same label are clustered together, and those with different labels are widely spaced [37].

When N training samples $x_{1}$ to $x_{N}\;$are taken from the underlying sub-manifold M. The between-class graph in LSDA is thus created by a vertex set X that is not specified as well as the weight matrix $W_{w}$. The subscription w displays the within-class subscription. The weight matrix elements $W_{w,\;ij}$ are shown below [38]:

(3) $$W_{w,\;ij}=\left\{\begin{array}{c} 1\;\;\;if\;x_{i}\in N_{w}\left(x_{j}\right)\;or\;x_{j}\;\in N_{w}\left(x_{i}\right)\;\\ 0\;\;\;\;\;\;\;\;\;\;\;\;\;\;\;\;\;\;\;\;\;\;\;\;\;\;\;\;\;\;\;\;\;\;\;\;\;otherwise \end{array}\right\}$$

where the set $N_{w}\left(x_{i}\right)$ consists of the k closest neighbors of $x_{i}$ which have the same label as $x_{i}$. Within-class compactness in LSDA may be learned via Laplacian embedding, as shown below:

(4) $$\min_{y}J\left(y\right)=\underset{i,j}{\sum }{\left(y_{i}-y_{j}\right)}^{2}W_{w,\;ij}$$

where $y_{i}=\alpha^{T}x_{i}$ is a scale and shows the one-dimensional map of $x_{i}.\alpha$ is the projection direction. To characterize the similarity of local data, the objective function (2) is often utilized. In this research, we employed LSDA to optimize class margins and improve classification performance. To that purpose, LDSA is used to decrease the retrieved characteristics of each sub-band [37]. In the following sections, the combination of four sets of extracted features from each sub-bands creates the feature vector.

### 2.3. Step Three: Classification

Finally, a deep neural network (DNN)-based classifier was trained with the reduced features. DNNs have demonstrated higher performance when compared to traditional ANNs and have found a variety of applications in image recognition. A complex network, on the other hand, necessitates high-performance computing capacity as well as a significant amount of time. To avoid using a computationally expensive and slow classifier, we developed a simple, rapid, fully connected-based classifier by decreasing features and improving discrimination with LDSA.

**Figure 3:**
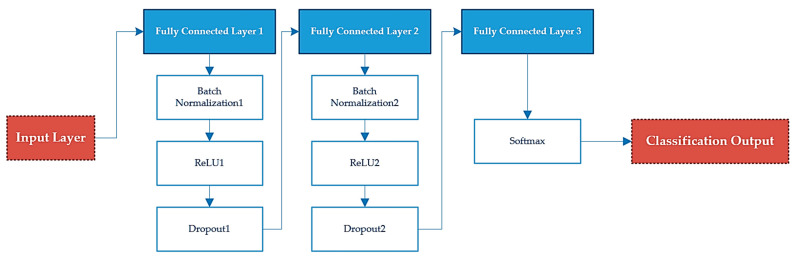
DNN Architecture.

According to LDSA-modified input features, the images were subsequently categorized using a simple DNN. After that, a fully connected (FC) network was utilized for classy crack and non-crack images. Batch normalization layers sped up the training process, while rectified linear unit (ReLU) and dropout were employed to reduce overfitting issues and generalization. Finally, a SoftMax layer composed of two nodes linked to the final FC divided images into two groups. The architecture of this DNN is depicted in Figure 3.

### 2.4. Reference Image Classification Models

LeCun et al. [39] were the first ones who applied CNN in computer vision applications. After years, CNNs became popular in other fields such as video analysis and face recognition object detection. CNNs are a sort of neural network that provides higher-resolution automatic feature extraction. The images are presented in three scales, from lowest to highest order. The first neural network extracts local characteristics and microscopic details such as edges, lines, and curves. The retrieved features are assembled at the subsequent deeper levels, and the final layers reconstruct the entire image. Convolution, pooling, fully connected layers, and activation functions are the primary processes in this procedure.

CNNs adopt two distinct training methodologies. The first is training the network from scratch, which requires massive amounts of data. The second is transfer learning using pre-trained models and parameter tuning. This method is more efficient and uses significantly fewer data for network training. Transfer learning involves training a network on a large dataset and then transferring the bias of the learned weights to another network with a new task. The vast majority of pre-trained networks were trained using the ImageNet dataset of one million pictures with one thousand categories [11].

Based on research by Nguyen et al. [40], eight of the popular networks for image classification were adapted, trained on concrete crack images, and compared in terms of performance and stability under adverse conditions. The networks were studied in two classes: smaller models (AlexNet, SqueezeNet, Goog-leNet, and ResNet-18) and larger models (ResNet-50, ResNet-101, InceptionV3, and Xception). The results demonstrated that GoogleNet (Small model) and Xception (Large model) were challenged and verified as highly reliable networks on average, particularly when utilized with reasonable batch sizes.

In this paper, the performance of the established method is compared with the pairs of small and large models GoogleNet and Xception, respectively. Other experiments have demonstrated their capacity to detect fractures and damage. Table 1 outlines the key attributes of these two models [40].

Using a non-adaptive learning rate approach called stochastic gradient descent with momentum (SGDM), the authors were able to optimize network parameters and boost network performance in terms of over-fitting, convergence, generalization, and runtime. L2 regularization was used across all layers to prevent over-fitting. The training was conducted over the length of 2700 iterations and 300 epochs. As a result of trial and error, the appropriate values were determined to guarantee convergence of training progress in the initial stages. To ensure that all the pre-trained models were being compared on a level field, we used the hyperparameters from Table 2. In order to achieve the best performance with the least amount of over-fitting, these settings were determined by a grid search based on prior study [40].

### 2.5. Indices of Evaluation

The classification efficiency of neural networks can be measured using several distinct indices. We employed three of the most commonly used metrics for image classification. In this context, precision refers to the accuracy with which a given result is identified, whereas recall measures the extent to which all relevant results are correctly predicted. One may also refer to the recall rate of a validation, which is another terminology for accuracy. When evaluating the efficacy of different CNNs, the F1-score is beneficial, since it integrates the accuracy and recall variables into a single, straightforward measure.

(5) $$\mathrm{Precision}\;=\frac{\mathrm{True}\;\mathrm{Positive}}{\mathrm{Actual}\;\mathrm{Classes}}$$

(6) $$\mathrm{Recall}=\frac{\mathrm{True}\;\mathrm{Positive}}{\mathrm{Predicted}\;\mathrm{Classes}}$$

(7) $$F1-\mathrm{score}=\;\frac{2\;\times \mathrm{Precision}\;\times \;\mathrm{Recall}}{\mathrm{Precision}+\mathrm{Recall}}$$

In order to have a better comparison in a view, we combined the indices into a new index named Overall F1Score, as below:

(8) $$\mathrm{Overall}\;F1-\mathrm{score}=\frac{2\times \mathrm{Overall}\;\mathrm{Precision}\;\times \;\mathrm{Overall}\;\mathrm{Recall}}{\mathrm{Overall}\;\mathrm{Precision}+\mathrm{Overal}\;\mathrm{Recall}}$$

where the overall term indicates the average values for each index.

### 2.6. Concrete Image Dataset

In several ways, the integrity of image data can be compromised after pictures are taken in practice. The appearance of salt and pepper owing to improper digital ISO settings, Gaussian noise due to image sensor temperature increase in voltage and illumination setbacks, and motion blur due to relative movement between the image-capturing equipment and focus sites are among the most typical reasons [28].

Thus, to construct two versions of the original 3000 image dataset, salt and pepper noise and motion blur are applied to the original data. These images were taken from the surfaces of concrete walls and have been cropped to 256 × 256 pixels. The variant sets are referred to as 3000 SP and 3000 MB image datasets in the rest of this investigation. A noise density of 6% is used for salt and pepper noise and motion blur, the motion duration is set to 20 pixels, and the motion angle is set to 11 degrees. Readers are invited to obtain the dataset and related details in these references [40,41].

**Figure 4:**
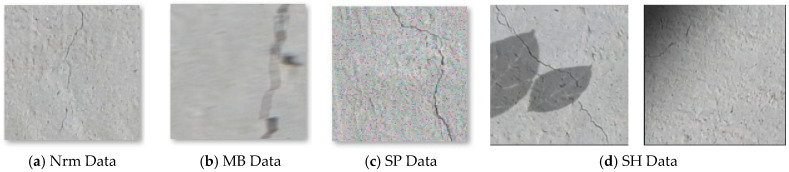
Normal Image data versus compromised variants.

Shadows and ambient lighting are the other adverse situations that occur in the actual world. So, different shadows, such as black rectangles, leaves, and gradient shadows, are applied on the original images to simulate real-world settings named SH data. Figure 4 depicts four types of image variation utilized to imitate adverse conditions in the current study.

In this study, each model is evaluated with the same 3000 image dataset in an approximate 70/15/15 ratio for training/validation/testing. The mentioned performance indices employ the test data that the networks have not previously seen.

## 3. Related Studies

Although wavelets and deep learning have been utilized in several studies for damage identification [4,42,43,44], few researchers have employed the combination of them to detect cracks in image datasets. In this section, a brief literature review is provided on the application of deep learning and wavelets to the detection of concrete crack image.

To overcome the issues of manual inspection, Dixit and Wagatsuma [45] proposed a novel method to detect micro-cracks in parts of concrete bridges using a drone with a high-resolution proximity camera. To study the texture features of the images of bridge deck, we used morphological component analysis (MCA) based on sparse coding. Dual tree complex wavelet transform (DTCWT) and anisotropic diffusion (AD) were used in MCA to find the coarse components of the images acquired by camera.

AlexNet, GoogleNet, SqueezNet, ResNet-18, ResNet-50, ResNet-101, DenseNet-201, and Inception-v3 were among the models used by Ranjbar [46] et al. to evaluate the performance of retrained deep learning models in the detection and classification of pavement cracking. In addition, a more efficient crack segmentation technique is provided, one that employs a modified wavelet transform module and extra regularization parameters. The results of the confusion matrix performance ranged from 0.94 to 0.99, indicating that retrained classifier models generate reliable results.

After conducting non-destructive ultrasonic testing on a structure, Arbaoui et al. [47,48] developed an effective way to track the development of cracks in the concrete. This technique relies on wavelet-based multi-resolution analysis of ultrasonic signals collected from samples or specimens of the examined material that have been subjected to a variety of different solicitation. Finally, the authors proposed a deep leaning-based method for crack detection using CNNs.

To address the challenge of accurately predicting the location of surface cracks in concrete, Yang et al. [49] offer a unique discrete wavelet transform and attention (DWTA) module built on top of the popular skeleton network U-net. Both the mean intersection and mean union over the mean F1-score are 0.848 and 0.824, respectively.

Based on the preceding discussion, this study attempts to not only boost classification speed by employing feature reduction, feature fusion, and a fully connected deep learning classifier, but also to improve accuracy, particularly in environmental adverse conditions, using wavelet transformation. According to the authors’ knowledge, this is the first method for concrete crack classification based on wavelet transformation and deep learning that surpasses typical pre-trained CNN models in terms of speed and accuracy and considering different adverse conditions.

## 4. Results and Discussion 

FastCrackNet’s performance is compared to that of the reference techniques in this section under a variety of conditions. Following that, the purpose and impact of its components, including transformation and feature reduction, are studied. Finally, the robustness is evaluated in the presence of different network designs, input data uncertainties and overfitting issues.

### 4.1. Efficiency Evaluation

Initially, FastCrackNet’s performance is assessed in a variety of adverse scenarios, employing the same number of epochs and total iterations (i.e., 2700 and 300) as GoogleNet and Xception. The comparison of efficiency begins with a comparison of classification performance and processing time. The proposed strategies are devised and implemented in MATLAB 2020b with the help of a deep learning package. This study used a ASUS VivoBook (ASUS, Fort Riley, KS, USA) with the following specifications, for all methods to have a fair comparison.

- AMD Ryzen 5 3500U Processor (4M Cache, 2.1 GHz up to 3.7 GHz);
- 24 GB DDR4 RAM & 512 GB SSD;
- Nvidia GTX 1050 4 GB Graphics.
  Nvidia GTX 1050 4 GB Graphics.

The suggested technique exceeds the previous reference methods in terms of classification accuracy for all variations, as indicated in Table 3. Furthermore, adverse conditions have nearly no effect on FastCrackNet’s efficiency. In terms of classification performance, our technique is almost 60 and 80 times quicker than GoogleNet and Xeption, respectively, and it was shown that our proposed method greatly improved the computing time.

### 4.2. Finding Time-Saving Approach

This section specifies a criterion for determining the most competent way to classify images in noisy circumstances, taking performance and calculation cost into account. The efficiency index (EI) is calculated using the following formula:

(9) $$EI=\frac{Pm}{CT}\times 100$$

where Pm is each method’s average performance as measured by its F1-score across all cases, and CT is the duration of time spent on the computations, presented in Table 3. This index provides an overall perspective of each methodology by averaging its performance and calculation time over three image types in both standard and noisy settings. Thus, better efficiency with less computing time corresponds to a higher EI value.

## 5. Stability Assessment

Herein, some of the typical uncertainties in the training of models are discussed. To this end, the stability and efficiency of the proposed method are being evaluated within four situations, namely change of bath size, input image size, the number of samples and generalization.

### 5.1. Batch Size Impact

Many hyperparameters must be modified in order to train deep learning architectures to classify images correctly. These parameters will have an influence on the network’s performance as it converges. Herein, the tuning size of batches, or the number of input images employed in each training epoch, is an important hyperparameter. If this factor is set too high, the network may take too long to converge. On the other hand, if it is set too low, the network may oscillate excessively, yielding undesirable results. Another variable that may influence batch size is the dataset’s complexity, which is especially considerable in medical data [50].

**Figure 5:**
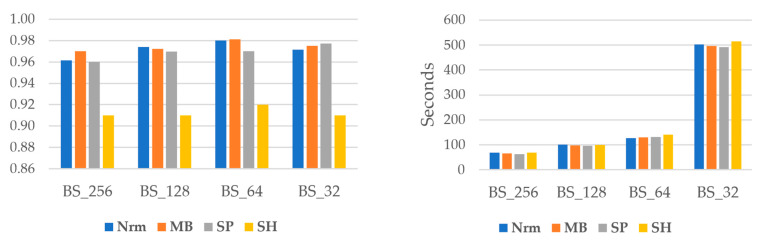
Comparison of overall F1Score and computation time.

The overall F1Score and computation time are shown in Figure 5. It is clear that when batch size declines, analysis time increases. For example, the necessary time for the original image is nearly seven times greater for a batch size of 32 than 256. Additionally, except for the SP data, the batch size of 64 gives better accuracy in all image variants.

**Figure 6:**
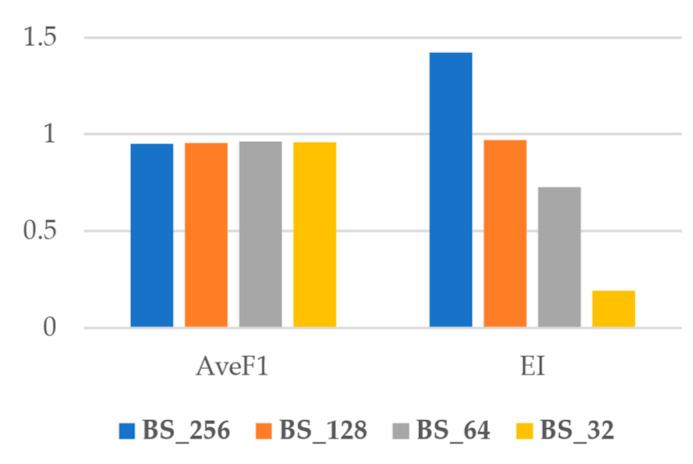
AveF1 and EI index for different batch sizes.

Figure 6 shows the average of the overall F1Score (AveF1) and EI for each batch size. As shown, while the AveF1 is nearly constant for all batches, the EI increases significantly from lower batch sizes to higher ones. Accordingly, we conclude that using higher batch sizes is more efficient as the computation time drops dramatically for higher batch sizes, but at the same time, the accuracy does not change.

### 5.2. Image Size Impact

The size of images can have an impact on training performance. As a result, in this section, we challenge the stability of the proposed approach. In the previous investigation of this paper, the input image was 64 × 64 pixels. In order to see the impact of input image size, in this section, the image data sets are resized into two different sets, including 48 × 48 and 56 × 56 pixels. Likewise, before, the classification was conducted via FastCrackNet, and the results are compared. 

**Figure 7:**
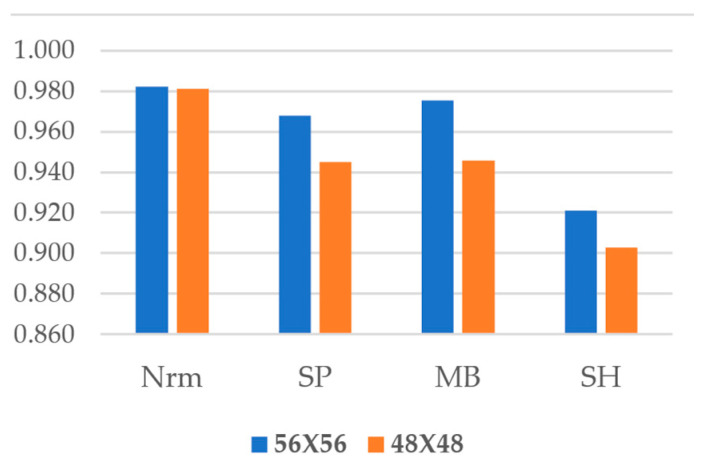
Comparison of Impact of Image Sizes.

As seen in Figure 7, the normal data experienced the least variance owing to sample changes and achieved almost the same level of accuracy. FastCracknet’s accuracy does not fall below 0.90 in either of the reduced scenarios.

### 5.3. Impact of Number of Samples 

To see the impact of samples in the performance of the network, herein, two conditions with different numbers of images for training are defined: one with 90% of the original data and the other with 80%. This includes 2700 and 2400 images, respectively, for the new dataset. Once again, the FastCrackNet classifies images but with a new number of images.

**Figure 8:**
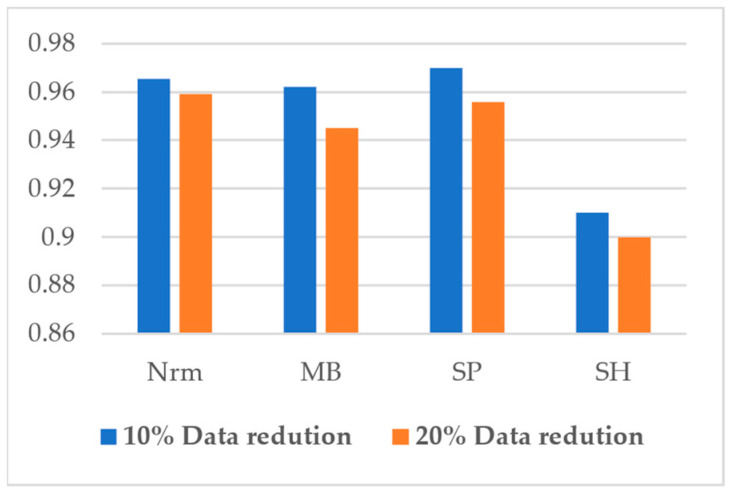
Impact of Data Reduction.

The results for the two scenarios are denoted in Figure 8. It is apparent that a reduction of 20% of tainting data could degrade the performance higher in all cases. Overall, the variations in all conditions fluctuate between nearly 0.90 to less than 0.97, which shows the stability of the model in terms of training samples.

### 5.4. Generalization

One of the main issues in the training of models is the problem of over-fitting, which occurs when a model learns too specifically from its input data. This could degrade the performance of the model by changing the input data. One way for evaluating the effectiveness of a machine learning model is to employ cross-validation, which is a resampling strategy used to evaluate a model when insufficient data is available. 

**Figure 9:**
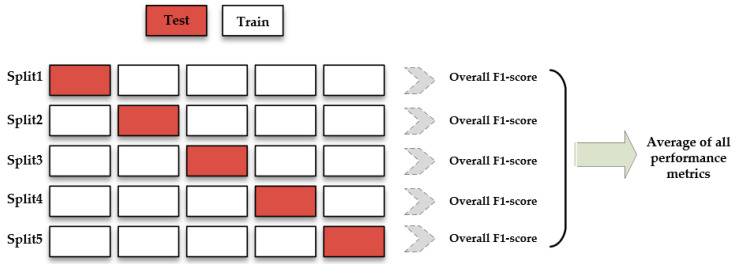
K-fold cross validation procedure.

Cross-validation necessitates preserving some data that were not used to train the model so that it may be tested and validated later. The input dataset is divided into K equal-sized subsets for the k-fold cross-validation method. The prediction function uses k-1 folds for each training set, keeping the rest folds for the test set. K-fold cross-validation separates the dataset into K distinct subsets (See Figure 9). The size of the data set determines the value of K, and one part of the dataset (out of k total) is utilized for testing, while the rest is used for training. 

In this research, k-fold cross-validation is conducted for K=10 folds, and the results are shown for test data in Table 4. The lack of noticeable changes in the accuracy reveals that the proposed technique is well-fitted for training data.

## 6. Conclusions

The major purpose of this research was to create a reliable and computationally efficient crack identification method for identifying pictures of concrete fractures obtained under varied unfavorable situations. Two types of digital filters were utilized to imitate unfavorable conditions in the images: motion blur, and salt and pepper noise. Because of the small cracks, these noise types were introduced to a complex concrete crack dataset, and the noise challenge was effectively handled using a unique deep learning-based image classification system named FastCrackNet.

The three computational steps of FastCrackNet are the wavelet image processing, the LSDA feature conditioner, and a basic 12-layer deep network for classification. In order to evaluate and compare FastCrackNet’s performance, two of the most renowned CNN models, Xception and GoogleNet, were employed to identify concrete cracks under various adverse conditions. It was revealed through a comparison of two methods and the use overall of F1-score data that:

1. FastCrackNet outperforms the traditional CNNs in terms of the accuracy of concrete crack classifications.
2. FastCrackNet does not degrade due to adverse environmental conditions.
3. FastCrackNet is significantly faster than the pre-trained CNNs.
4. The batch size and uncertainties do not affect the performance of FastCrackNet significantly.
5. The proposed approach encounters no over-fitting issues.

## Figures and Tables

**Table 1:** Characteristics of the two networks used for comparison.

Network	Layers	Size (MB)	Parameters	Input Image Size
GoogleNet	22	27	7.0	224 × 224
Xception	71	85	22.9	229 × 229

**Table 2:** Hyper-parameters of networks.

Parameter	Value
Optimization method	SGDM
Initial learning rate	$1e^{-4}$
L2 Regularization	$1e^{-3}$
Epochs	300
Iterations	2700

**Table 3:** Comparison of Methods.

Model	Image Type	Overall F1-Score	Time (s)
**GoogleNet**	Nrm	0.95	8635
	SP	0.93	8203
	MB	0.92	8191
	SH	0.86	8251
**Xception**	Nrm	0.97	11,235
	SP	0.93	10,662
	MB	0.92	10,591
	SH	0.84	11,657
**FastCrackNet**	Nrm	0.98	127
	SP	0.98	130
	MB	0.97	132
	SH	0.92	141

**Table 4:** K-fold cross-validation results.

Model	Image Type	Average of Overall F1-Score
**FastCrackNet**	Nrm	0.95
	SP	0.94
	MB	0.95
	SH	0.90

## Data Availability

The data presented in this article are available upon request to the corresponding authors.

## References

[1] Affonso C., Rossi A.L.D., Vieira F.H.A., de Leon Ferreira A.C.P. (2017). Deep learning for biological image classification. Expert Syst. Appl..

[2] Hao S. (2010). I-35W bridge collapse. J. Bridge Eng..

[3] Behnam B. (2019). Fire structural response of the plasco building: A preliminary investigation report. Int. J. Civ. Eng..

[4] Gharehbaghi V.R., Noroozinejad Farsangi E., Noori M., Yang T., Li S., Nguyen A., Málaga-Chuquitaype C., Gardoni P., Mirjalili S. (2021). A critical review on structural health monitoring: Definitions, methods, and perspectives. Arch. Comput. Methods Eng..

[5] Spencer B.F., Hoskere V., Narazaki Y. (2019). Advances in computer vision-based civil infrastructure inspection and monitoring. Engineering..

[6] Graves A. (2012). Long short-term memory. Supervised Seq. Label. Recurr. Neural Netw..

[7] Gu J., Wang Z., Kuen J., Ma L., Shahroudy A., Shuai B., Liu T., Wang X., Wang G., Cai J. (2018). Recent advances in convolutional neural networks. Pattern Recognit..

[8] Hinton G.E. (2009). Deep belief networks. Scholarpedia.

[9] Vincent P., Larochelle H., Bengio Y., Manzagol P.-A. Extracting and composing robust features with denoising autoencoders. Proceedings of the 25th international Conference on Machine learning.

[10] Medsker L.R., Jain L. (2001). Recurrent neural networks. Des. Appl..

[11] Krizhevsky A., Sutskever I., Hinton G.E. (2017). Imagenet classification with deep convolutional neural networks. Commun. ACM.

[12] Fujita Y., Hamamoto Y. (2011). A robust automatic crack detection method from noisy concrete surfaces. Mach. Vis. Appl..

[13] Du G., Cao X., Liang J., Chen X., Zhan Y. (2020). Medical image segmentation based on u-net: A review. J. Imaging Sci. Technol..

[14] Jenkins M.D., Carr T.A., Iglesias M.I., Buggy T., Morison G. A deep convolutional neural network for semantic pixel-wise segmentation of road and pavement surface cracks. Proceedings of the 2018 26th European signal processing Conference (EUSIPCO).

[15] Minaee S., Boykov Y.Y., Porikli F., Plaza A.J., Kehtarnavaz N., Terzopoulos D. (2021). Image segmentation using deep learning: A survey. IEEE Trans. Pattern Anal. Mach. Intell..

[16] Galvez R.L., Bandala A.A., Dadios E.P., Vicerra R.R.P., Maningo J.M.Z. Object detection using convolutional neural networks. Proceedings of the TENCON 2018-2018 IEEE Region 10 Conference.

[17] Wu X., Sahoo D., Hoi S.C. (2020). Recent advances in deep learning for object detection. Neurocomputing.

[18] Zhao Z.-Q., Zheng P., Xu S.-t., Wu X. (2019). Object detection with deep learning: A review. IEEE Trans. Neural Netw. Learn. Syst..

[19] Zhu J., Song J. (2020). An intelligent classification model for surface defects on cement concrete bridges. Appl. Sci..

[20] Hossain M.Z., Sohel F., Shiratuddin M.F., Laga H. (2019). A comprehensive survey of deep learning for image captioning. ACM Comput. Surv. (CsUR).

[21] Wang C., Yang H., Bartz C., Meinel C. Image captioning with deep bidirectional LSTMs. Proceedings of the 24th ACM International Conference on Multimedia.

[22] Dung C.V. (2019). Autonomous concrete crack detection using deep fully convolutional neural network. Autom. Constr..

[23] Islam M.M., Hossain M.B., Akhtar M.N., Moni M.A., Hasan K.F. (2022). CNN Based on Transfer Learning Models Using Data Augmentation and Transformation for Detection of Concrete Crack. Algorithms.

[24] Ma D., Fang H., Wang N., Xue B., Dong J., Wang F. (2022). A real-time crack detection algorithm for pavement based on CNN with multiple feature layers. Road Mater. Pavement Des..

[25] Zhang C., Nateghinia E., Miranda-Moreno L.F., Sun L. (2022). Pavement distress detection using convolutional neural network (CNN): A case study in Montreal, Canada. Int. J. Transp. Sci. Technol..

[26] Chen L.-C., Papandreou G., Kokkinos I., Murphy K., Yuille A.L. (2017). Deeplab: Semantic image segmentation with deep convolutional nets, atrous convolution, and fully connected crfs. IEEE Trans. Pattern Anal. Mach. Intell..

[27] Wu L., Lin X., Chen Z., Lin P., Cheng S. (2021). Surface crack detection based on image stitching and transfer learning with pretrained convolutional neural network. Struct. Control. Health Monit..

[28] Chianese R., Nguyen A., Gharehbaghi V., Aravinthan T., Noori M. (2021). Influence of image noise on crack detection performance of deep convolutional neural networks. arXiv.

[29] Rader C., Brenner N. (1976). A new principle for fast Fourier transformation. IEEE Trans. Acoust. Speech Signal Process..

[30] Griffin D., Lim J. (1984). Signal estimation from modified short-time Fourier transform. IEEE Trans. Acoust. Speech Signal Process..

[31] Johansson M., The Hilbert Transform Master’s Thesis. Växjö University, Suecia. http://w3.msi.vxu.se/exarb/mj_ex.pdf.

[32] Gharehbaghi V.R., Kalbkhani H., Noroozinejad Farsangi E., Yang T., Nguyen A., Mirjalili S., Málaga-Chuquitaype C. (2022). A novel approach for deterioration and damage identification in building structures based on Stockwell-Transform and deep convolutional neural network. J. Struct. Integr. Maint..

[33] Sutha S., Leavline E.J., Singh D. (2013). A comprehensive study on wavelet based shrinkage methods for denoising natural images. WSEAS Trans. Signal Process..

[34] Parida P., Bhoi N. (2017). Wavelet based transition region extraction for image segmentation. Future Comput. Inform. J..

[35] Rinky B., Mondal P., Manikantan K., Ramachandran S. (2012). DWT based feature extraction using edge tracked scale normalization for enhanced face recognition. Procedia Technol..

[36] Yelampalli P.K.R., Nayak J., Gaidhane V.H. (2018). Daubechies wavelet-based local feature descriptor for multimodal medical image registration. IET Image Process..

[37] Cai D., He X., Zhou K., Han J., Bao H. Locality sensitive discriminant analysis. Proceedings of the IJCAI.

[38] Attoui I., Fergani N., Boutasseta N., Oudjani B., Deliou A. (2017). A new time–frequency method for identification and classification of ball bearing faults. J. Sound Vib..

[39] LeCun Y., Bengio Y., Hinton G. (2015). Deep learning. Nature.

[40] Nguyen A., Chianese R.R., Gharehbaghi V.R., Perera R., Low T., Aravinthan T., Yu Y., Samali B., Guan H., Khuc T., Guan H., Chan T.H.T. (2022). Robustness of Deep Transfer Learning-Based Crack Detection against Uncertainty in Hyperparameter Tuning and Input Data. Recent Advances in Structural Health Monitoring Research in Australia.

[41] (2021). 3000_ImageData_for_Crack_Detection; Kaggle. https://www.kaggle.com/datasets/nguyen49/3000-imagedata-for-crack-detection.

[42] Rafiei M.H., Adeli H. (2018). A novel unsupervised deep learning model for global and local health condition assessment of structures. Eng. Struct..

[43] Ai L., Soltangharaei V., Ziehl P. (2021). Evaluation of ASR in concrete using acoustic emission and deep learning. Nucl. Eng. Des..

[44] Gharehbaghi V.R., Farsangi E.N., Yang T., Hajirasouliha I. (2021). Deterioration and damage identification in building structures using a novel feature selection method. Structures.

[45] Dixit A., Wagatsuma H. Comparison of effectiveness of dual tree complex wavelet transform and anisotropic diffusion in MCA for concrete crack detection. Proceedings of the 2018 IEEE International Conference on Systems, Man, and Cybernetics (SMC).

[46] Ranjbar S., Nejad F.M., Zakeri H. (2021). An image-based system for pavement crack evaluation using transfer learning and wavelet transform. Int. J. Pavement Res. Technol..

[47] Arbaoui A., Ouahabi A., Jacques S., Hamiane M. (2021). Wavelet-based multiresolution analysis coupled with deep learning to efficiently monitor cracks in concrete. Frat. Ed Integrità Strutt..

[48] Arbaoui A., Ouahabi A., Jacques S., Hamiane M. (2021). Concrete cracks detection and monitoring using deep learning-based multiresolution analysis. Electronics.

[49] Yang G., Geng P., Ma H., Liu J., Luo J. DWTA-Unet: Concrete Crack Segmentation Based on Discrete Wavelet Transform and Unet. Proceedings of the 2021 Chinese Intelligent Automation Conference.

[50] Kandel I., Castelli M. (2020). The effect of batch size on the generalizability of the convolutional neural networks on a histopathology dataset. ICT Express.

